# The Mediator Complex: A Regulatory Hub for Transcriptional Activity of Nuclear Receptors

**DOI:** 10.3390/cells14171335

**Published:** 2025-08-28

**Authors:** Liming Zhou, Manhan Zhao, Yifei Zhai, Qiong Lin

**Affiliations:** School of Medicine, Jiangsu University, 301 Xuefu Road, Zhenjiang 212013, China; 2212313098@stmail.ujs.edu.cn (L.Z.); 2212313096@stmail.ujs.edu.cn (M.Z.); 2212413005@stmail.ujs.edu.cn (Y.Z.)

**Keywords:** Mediator complex, nuclear receptors, transcriptional regulation, protein interactions, the Mediator-associated diseases

## Abstract

The Mediator complex plays a key role in gene transcription. In particular, the interaction of the Mediator complex with nuclear receptors, the known transcription factors, regulates multiple nuclear receptor-mediated gene transcription pathways and associated cellular functions. Dysregulation of the interaction of the Mediator complex with nuclear receptors results in many pathological processes, such as cancer, metabolic and neuronal diseases. Thus, understanding of the mechanism by which the Mediator complex regulates the nuclear receptor-mediated transcriptional activity and biological function is crucial for therapy of both the Mediator complex- and nuclear receptor-associated diseases. In this review article, we attempt to summarize current research progress in the interaction of the Mediator complex with nuclear receptors and the associated nuclear receptor transcriptional signaling pathways, explore the clinical potential of the Mediator complex as a therapeutic target, and provide new perspectives for the treatment of diseases associated with the Mediator complex and nuclear receptors.

## 1. Overview of the Mediator Complex

The Mediator complex is an evolutionarily conserved multi-subunit protein complex and plays a key role in the RNA polymerase II (Pol II)-mediated gene transcription [1]. The Mediator complex was first discovered by Dr. Roger Kornberg during his study on the gene transcription mechanism of Saccharomyces cerevisiae and was found to function in transcriptional activation [2,3]. The Mediator complex, from yeast to humans, is highly conserved structurally and functionally in the transcriptional regulation of all eukaryotes [4]. The Mediator complex transmits transcriptional regulatory signals through its subunit modules, which interact with both RNA Pol II and transcription factors (TFs) and achieve the regulation of gene expression [5].

### 1.1. Composition and Structure of the Mediator Complex

**Figure 1:**
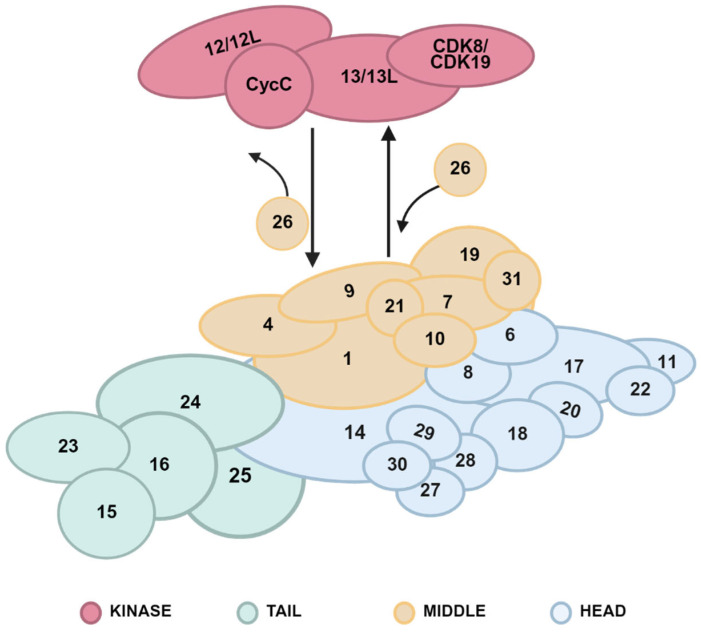
Composition of the human Mediator complex. The human Mediator complex contains four modules: head (light blue), middle (light yellow), tail (light green) and the dissociable CDK8 kinase (red) modules. The head, middle and tail modules constitute the core of the Mediator complex. MED14 scaffolds other subunits, thereby stabilizing the complex. The tail module’s variability facilitates adaptation to diverse transcriptional regulatory requirements. The CDK8 kinase module reversibly binds to the Mediator complex core and its binding competes with MED26 [6]. The digital labels in the figure correspond directly to the subunit names.

The Mediator complex is a large protein complex and contains multiple subunits. The complex can be structurally divided into four modules: the head module, the middle module, the tail module, and a dissociable CDK8 kinase module (Figure 1 and Table 1) [6,7]. The human Mediator complex subunits were named by following those of the homologues in yeast. In human cells, the Mediator complex core, including the head, the middle and the tail modules, comprises 26 subunits. Upon binding of the CDK8 kinase module to the core of the Mediator complex, the subunit MED26 is dissociated. Thus, the CDK8 kinase module-contained-Mediator complex has 29 subunits. The binding of the CDK8 module to the Mediator complex core is a dynamic and reversible process [6,7] (Figure 1). Despite structural and functional conservation of the Mediator complex between yeast and human, several subunits in the human Mediator complex, such as MED23 through MED30, have not been found in yeast, suggesting more complex regulation in human genome transcriptional processes.

The head and middle modules constitute a minimal structural and functional core of the Mediator complex and interact with RNA Pol II. Among the subunits, MED14 is a unique subunit in the Mediator complex with a key role in scaffolding other subunits, thus maintaining the interaction and stabilization of the Mediator complex modules [8]. Currently, which module MED14 belongs to is still an area of dispute. Here, we consider MED14 as a subunit of the head module based on the comment proposed by Abdella R et al. [9]. The tail module comprises subunits that interact with transcriptional activators and repressors and displays significant variability, a characteristic for adaptation to diverse transcriptional regulatory demands through conformational changes [10]. The CDK8 kinase module comprises four subunits: cyclin C (CycC), CDK8/CDK19, MED12/MED12L, and MED13/MED13L. This module serves as a molecular switch that both hinders the binding of the Mediator complex to RNA Pol II and phosphorylates the C-terminal domain (CTD) of RNA Pol II by CDK8, thereby inhibiting transcription initiation and transcription elongation [11]. However, in hypoxic response, this module also functions to facilitate transcription elongation [12].

### 1.2. The Function of the Mediator Complex

The Mediator complex plays a pivotal role in eukaryotic gene expression, with its function extending across transcription initiation, elongation, termination, mRNA processing, and the activation of non-coding RNAs. The Mediator complex’s ability to integrate diverse transcriptional regulatory signals guarantees normal gene expression and cell homeostasis. Dysregulation of its function results in abnormality of gene transcription and pathogenesis and progression of various diseases.

During the transcription initiation phase, the Mediator complex serves as a central component of the pre-initiation complex (PIC), linking transcription factors with RNA Pol II. Through its head, middle, and tail modules, the Mediator complex interacts with various PIC components, facilitating the assembly and stabilization of the PIC. Furthermore, through interacting with the CTD of RNA Pol II, the Mediator complex promotes phosphorylation of RNA Pol II and activates transcription [13]. The Mediator complex coordinates transcription factors with RNA Pol II during the initiation and elongation phases of transcription. For instance, the Mediator complex enhances transcriptional activity by directly interacting with TFIIB and RNA Pol II, facilitating the recruitment of TFIIB to and strengthening the association of RNA Pol II with the promoter [8]. The subunit MED26 serves as a docking site for transcription elongation factors by its N-terminal domain, assembles the transcription elongation factor complexes, and promotes the transition of RNA Pol II from the initiation phase to the elongation phase of transcription [14]. At the same time, the Mediator complex also participates in the transcription termination process through the subunit MED26 by recruiting the little elongation complex (LEC), thereby facilitating the efficient transcription termination of RNA Pol II [15].

The Mediator complex does not always promote transcription. It represses transcription with its CDK8 kinase module. The CDK8 kinase module, a dissociable component of the Mediator complex, exerts transcriptional repression through multiple mechanisms. Its kinase activity can phosphorylate the CTD of RNA Pol II or other transcription factors and spatially obstruct the interaction between the Mediator complex and RNA Pol II [16], thereby disrupting the assembly of the PIC and inhibiting transcription initiation and elongation. Furthermore, this module also facilitates transcription elongation. During hypoxic response, the CDK8 kinase module is recruited by HIF1A, the key transcription factor of the cellular response to hypoxia, subsequently attracting the super elongation complex (SEC) and CDK9, collectively promoting the transition of RNA Pol II from a paused state to an efficient elongation phase [12].

## 2. The Mediator Complex and Nuclear Receptors

**Figure 2:**
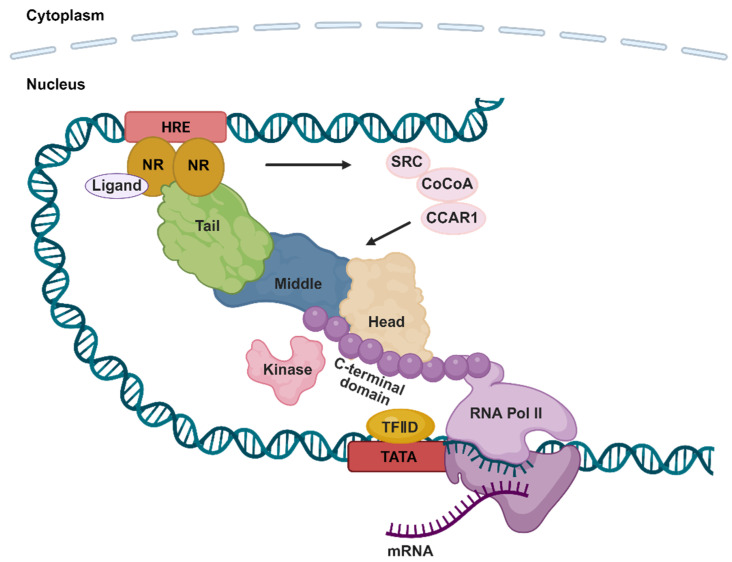
A model depicting the Mediator complex–nuclear receptor-mediated transcriptional activation. Upon activation by ligands and transcriptional initiation signals, nuclear receptors undergo conformational changes and recruit co-activators (e.g., SRC, CoCoA, etc.). Subsequently, co-activators mediate the recruitment of the Mediator complex. Following the Mediator recruitment, the CDK8 kinase module dissociates from the Mediator core, leading to binding of the Mediator complex to the RNA Pol II CTD and transcription factors. Thus, the Mediator complex serves as a “molecular bridge” to link nuclear receptors to transcription factors and RNA Pol II, promotes the transcriptional initiation by stabilizing the PIC assembly, and activates the nuclear receptor target gene expression.

Nuclear receptors are intracellular transcription factors that modulate the expression of genes associated with reproduction, development, and metabolism by binding to specific ligands, thereby linking signaling molecules to transcriptional responses [17]. Structurally, nuclear receptors comprise four functional domains: the N-terminal domain (NTD), the highly conserved DNA-binding domain (DBD), the C-terminal ligand-binding domain (LBD), and the hinge region that connects the DBD and LBD [18]. The LBD is the pivotal functional region of nuclear receptors, encompassing the activation function 2 (AF2) domain. It is responsible for recognizing and binding endogenous or exogenous ligands, thereby modulating downstream gene expression through conformational alterations [19]. Nuclear receptors regulate gene expression by binding to specific DNA sequences and recruiting various transcriptional co-regulators in a ligand-dependent manner. In the absence of transcription initiation signals or ligands, these receptors typically form complexes with heat shock proteins (such as HSP90), preventing their translocation into the nucleus. Alternatively, they associate with corepressors such as N-CoR/SMRT, which facilitates the recruitment of auxiliary factors like histone deacetylases (HDACs), promoting chromatin condensation and thereby repressing gene expression [17,20]. The molecular mechanisms by which nuclear receptors regulate target gene expression involve a dynamic, multi-step assembly of co-activator complexes [21]. Specifically, upon stimulation by ligand binding, nuclear receptors undergo conformational changes, dissociate from corepressors, and expose the AF1/AF2 domains that recruit co-activators (e.g., SRC1, CoCoA, etc.) that have histone acetyltransferase activity. These co-activators relax nucleosome structures to provide an accessible chromatin environment for transcription factors and RNA Pol II, and further recruit the Mediator complex (Figure 2). Complexes like SWI/SNF and RSC utilize the energy from ATP hydrolysis to reposition nucleosomes, facilitating easier access of DNA to transcription initiation sites [22]. In summary, these activated nuclear receptor co-activator complexes enable chromatin remodeling and coordinate the assembly and activation of the PIC, ultimately leading to the initiation of transcription.

The mammalian Mediator was initially isolated as the TRAP complex and was regarded as a co-activator of the thyroid hormone receptor (TR) [23]. Later, more evidence showed that the Mediator complex can directly engage in the regulation of both steroidal and non-steroidal nuclear receptors by enhancing their transcriptional activities, indicating that the Mediator complex is a general activator of nuclear receptors [4]. Nowadays, it is known that the Mediator complex directly interacts with nuclear receptors, serves as a bridge between nuclear receptors and basic transcriptional machinery, and regulates the nuclear receptor-mediated gene expression upon the ligand activation of nuclear receptors [24,25,26,27] (Figure 2). As shown in Table 2, MED1 is the subunit of the Mediator complex primarily involved in the regulation of nuclear receptor signaling. In addition, other subunits, such as MED13, MED14, MED15 and MED25, also participate in the regulation of the nuclear receptor signaling (Table 2). Notably, mutations or aberrant expression of Mediator complex subunits are associated with various diseases. For instance, the dysregulation of MED1 is prevalent in breast and prostate cancers, underscoring its pivotal role in nuclear receptor-associated cancers [28]. The specific functions of most Mediator complex subunits in nuclear receptor signaling remain largely unknown at present, particularly in terms of tissue specificity and dynamic regulation, which require further exploration.

In summary, as shown in Figure 2, the Mediator complex integrates nuclear receptor signals and transmits activation signals to the core promoter region, thereby driving the formation of the PIC and ensuring the efficient progress of transcription [27].

## 3. Interaction and Regulation of Nuclear Receptors by the Mediator Complex

### 3.1. Head Module

The head module of the Mediator complex contains 12 subunits (Figure 1 and Table 1) and mainly interacts with RNA Pol II through dynamic conformational rearrangements to regulate its transcriptional activity [43].

As the core subunit of the Mediator complex, MED14 plays a dual structural and functional regulatory role in the nuclear receptor signaling pathway. By directly binding to the N-terminal AF1 domain of nuclear receptors, MED14 activates their transcription activity without ligands [29,30]. It has been shown that MED14 interacts with glucocorticoid receptor (GR) and proliferator-activated receptor gamma (PPARγ) for assembly of the nuclear receptor transcriptional complexes and regulates cellular lipid homeostasis [29,30]. The mechanism underlying the interaction of MED14 with the nuclear receptors GR and PPARγ is very unique, as MED14 lacks the LXXLL motif, the known nuclear receptor interactive motif, indicating that MED14 promotes transcriptional activity of nuclear receptors through the non-LXXLL motif-mediated interaction.

### 3.2. Middle Module

The middle module of the Mediator complex comprises nine subunits (Figure 1 and Table 1). This module interacts with the head and tail modules to stabilize the structure of the Mediator complex and confers high flexibility. MED1, also known as thyroid hormone receptor-associated protein (TRAP220), is the subunit in the middle module best known for interaction with nuclear receptors and regulation of the nuclear receptor-mediated transcriptional processes.

As an integral component of the Mediator complex, MED1 directly interacts with nuclear receptors to establish a multifunctional transcriptional regulatory platform, thereby transducing the transcriptional signaling from nuclear receptors to RNA Pol II. It acts as a coactivator of nuclear receptors and is capable of enhancing the nuclear receptor transcriptional activity [36].

MED1 binds to the activation function 2 (AF2) region of the LBD domain of nuclear receptors via its conserved LXXLL nuclear receptor binding motif [44]. This mechanism has been rigorously validated in archetypal nuclear receptors, including the estrogen receptors (ER) and TR [45]. The interaction of MED1 with nuclear receptors is contingent upon the presence of a ligand. The ligand-bound nuclear receptors engage with MED1 to form a nuclear receptor–MED1 complex. MED1 in the complex recruits additional coactivators and activates transcription of specific target genes [23]. For instance, MED1 binds to TRβ via the LXXLL motif in the presence of thyroid hormone (T3) and enhances the transcription of the TRβ target gene *TSHB* [46]. During mammary gland development, MED1 also regulates ERα-dependent gene expression by binding to the ligand-activated ERα [26]. During adipogenesis, MED1 interacts with PPARγ via its two LXXLL motifs, modulating the expression of genes associated with adipogenesis and metabolism [47]. The interaction of MED1 with nuclear receptors is not limited to TRβ, ER, or PPARγ, as it was observed with GR [36], androgen receptors (AR) [33], and vitamin D3 receptors (VDR) [44]. The interaction of MED1 with all nuclear receptors is through the LXXLL-AF2 binding mode.

Studies indicate that nuclear receptors exhibit distinct binding preferences to the two LXXLL motifs of MED1 [48]. Typically, steroid hormone receptors, such as the ER, preferentially bind to the first LXXLL motif to activate target gene transcription [49]. In contrast, non-steroid hormone receptors like TRα and VDR modulate gene expression by interacting with the second LXXLL motif [50]. The preference in the binding to the LXXLL motifs of MED1 confers the distinct function of nuclear receptors. Specifically, ER primarily modulates hormone-associated breast cellular processes, while TRα and VDR are in non-hormonal pathways, such as calcium homeostasis and skeletal integrity [26,38,44].

Unlike most nuclear receptors, MED1 interacts with AR via an atypical α1/α2 helix/Tau-1 interactive mode in addition to the LXXLL/AF2 mode [51]. The Tau-1 is an important functional domain located at the N-terminus of AR. Upon ligand binding, Tau-1 interacts with co-activators to enhance the transcriptional activity of AR [51]. The 505–635 residue segment of MED1 (the RBD-1 region) that contains two α-helices (α1: 505–523; α2: 532–550) has been identified as the region interacting with the Tau-1 domain [33]. The α1/α2 structure forms a binding interface that directly anchors the Tau-1 domain [44]. The AR mutant lacking the Tau-1 domain completely loses its binding ability with MED1, while the mutant lacking the Tau-5 domain (another activation domain in AR) retains the binding activity [44]. Furthermore, the intra-molecular interaction between the N/C-terminal domains of AR, i.e., the N-terminal FXXLF motif, binds to the AF2 region, enhances the binding to MED1 by stabilizing the structure of Tau-1. When the N/C interaction is disrupted, the binding to MED1 is significantly reduced. It seems that the two LXXLL motifs of MED1 are not essential for AR binding [33].

### 3.3. Tail Module

The tail module contains five subunits (Figure 1 and Table 1) and acts as a bridge between a variety of transcription factors and RNA Pol II, enabling RNA Pol II to start the transcriptional process upon activation by transcriptional activators [6]. The subunits MED15, MED23, and MED25 in the tail module have been shown to interact with the nuclear receptors or nuclear receptor-coactivators and function in nuclear receptor signaling pathways.

#### 3.3.1. Interaction of MED15 with Sterol Regulatory Element-Binding Protein 1α (SREBP1α) in the Nuclear Receptor-Regulated Lipid Homeostasis

MED15 was initially identified as a binding protein of the mammalian sterol regulatory element-binding protein 1α (SREBP1α) [52]. Its KIX domain specifically interacts with the activation domain of SREBP1α, thereby enhancing the transcriptional activity of SREBP1α and facilitating expression of the genes for regulating the biosynthesis of cholesterol and fatty acids [4,53]. Studies have shown that MED15 knockdown leads to a decrease in expression of the SREBP-1 target genes, suggesting that MED15 plays a key role in SREBP-1-mediated gene expression and lipid biosynthesis [40]. Although SREBP-1α is not a nuclear receptor, it interacts with nuclear receptors to co-regulate gene expression and lipid metabolism and energy homeostasis. For instance, it synergizes with PPARγ, a pivotal regulator of adipocyte differentiation and maturation, to enhance expression of adipogenic genes [54]. Additionally, SREBP-1α interacts with liver X receptor (LXR), which plays a central role in cholesterol absorption and excretion, to modulate expression of the genes involved in cholesterol metabolism [55,56]. Thus, MED15 participates in the regulation of the expression of lipid biosynthesis genes through co-activation of SREBP-1α and nuclear receptors.

Interestingly, in *Caenorhabditis elegans*, the MED15 homologue MDT-15 interacts with the LBD of NHR-49 via the KIX domain and regulates fatty acid metabolism [39,57]. Knockdown of MDT-15 using RNA interference technology reduces the mRNA level of NHR-49 target genes in vivo [57,58]. In addition, the metal stress conditions enhance the binding of MDT-15 to the nuclear receptor HIZR-1 that protects the worm from the stress induced by high zinc or cadmium. MED15 in mammals also regulates the transcription of cadmium and zinc-responsive genes [59], suggesting that MED15 may interact with the HIZR-1-like nuclear receptor to mitigate metal stress in mammals.

Taken together, these findings underscore the critical role of MED15 in lipid homeostasis and cellular metal stress resilience via interaction with SREBP-1α and/or nuclear receptors.

#### 3.3.2. The Role of MED23 in Hepatic Fibrosis by Interaction with the Nuclear Receptor Retinoic Acid Receptor-Associated Orphan Receptor α (RORα)

MED23 serves as a crucial regulator of cellular energy homeostasis. Knockout of MED23 in the liver prevents obesity induced by high-fat diets or genetic predispositions and mitigates the associated complications in animal studies [60]. It has been shown that RORα directly transcribes the Ccl5 and Cxcl10 genes through its response element REORE that is associated with liver fibrosis [61,62]. MED23 interacts with RORα and inhibits the RORα transcriptional activity, thereby inhibiting expression of the Ccl5 and Cxcl10 genes [63]. MED23 knockout significantly upregulated the expression of Ccl5 and Cxcl10 in hepatocytes [63]. In a Ccl4-induced hepatic fibrosis murine model, specific knockout of MED23 in stem cells resulted in exacerbated liver fibrosis, accompanied by increased chemokine production and inflammatory infiltration [63]. These findings indicate that MED23 serves as a pivotal regulator in hepatic fibrosis by interacting with the nuclear receptor RORα.

#### 3.3.3. The Role of MED25 in the Orphan Nuclear Receptor Hepatocyte Nuclear Factor 4α (HNF4α)-Driven Insulin Secretion

MED25 binds to nuclear receptors through its C-terminal LXXLL motif. MED25 interacts with RAR and significantly activates the RAR/RXR heterodimer-mediated transcription [40]. Similarly, MED25 interacts with the HNF4α in a ligand-independent manner and modulates the HNF4α-mediated transcription of the genes associated with insulin secretion in pancreatic β-cells [64]. Furthermore, the interaction of MED25 with HNF4α is crucial for the normal development and function of pancreatic β-cells. The ligand-independent activation of HNF4α by MED25 may represent a unique intrinsic activation mechanism of HNF4α without external signals [64,65]. HNF4α is extensively expressed in the liver, kidneys, intestines, and pancreas and serves as a pivotal regulatory factor in the early development of pancreatic β-cells [66]. Functionally defective mutation of HNF4α causes the maturity-onset diabetes of the young (MODY), which is characterized by pancreatic β-cell dysfunction and impaired insulin secretion [65,67]. Mutations at the HNF4α binding site of MED25 are often associated with MODY, underscoring the role of MED25 in the HNF4α-mediated insulin secretion and associated diabetes [64].

In addition to regulating of expression of the genes associated with insulin secretion, MED25 also modulates the expression of the HNF4α target genes such as PPARα, L-type pyruvate kinase (L-PK), and glucose transporter 2 (GLUT2) by interacting with HNF4α, thereby exerting multidimensional control over drug metabolism and lipid homeostasis [41]. In the MIN6 pancreatic β-cell model, overexpression of MED25 significantly elevates the expression levels of these HNF4α target genes, while knockout of MED25 results in a sharp decline in the expression of these genes [64]. Mutation in the LXXLL motif of MED25, such as M364R, impairs its binding to HNF4α, and leads to transcriptional repression of these HNF4α target genes and inhibition of insulin secretion [64].

In summary, MED25 acts as a molecular hub in the HNF4α signaling pathway and plays a central role in insulin secretion of β-cells. Its functional aberration causes pathogenesis and progression of metabolic diseases such as MODY.

### 3.4. CDK8 Kinase Module

The kinase module of the Mediator complex consists of CDK8/CDK19, cyclin C, MED12/MED12L, and MED13/MED13L. This module reversibly associates with the core complex of the Mediator complex, serving as a ‘molecular switch’. Through dynamic binding to the core complex, integration of transcriptional signals, and phosphorylation of Pol-II, the CDK8 kinase module plays a central role in inhibition of transcription initiation, activation of transcription elongation, chromatin modification, and disease pathogenesis [68,69]. The subunits MED12, MED13 and CDK8 in the CDK8 module have been reported in nuclear receptor signaling pathways.

#### 3.4.1. Multifaceted Regulatory Roles of MED12 in Nuclear Receptor Signaling

MED12 is a pivotal subunit of the kinase module and maintains the structural integrity of the entire module [70,71]. Recent studies have identified MED12 as a regulator in AR signaling. Overexpression of MED12 is highly prevalent in castration-resistant prostate cancer (CRPC) and promotes tumor progression by enhancing AR activity [72]. AR-V7 is a splicing variant that lacks the ligand-binding domain but retains the DNA-binding domain. Thus, AR-V7 activates transcription of its target genes independent of androgen [73]. The expression of AR-V7 is associated with the CRPC, allowing the cancer cells to acquire the ability to resist the treatment with traditional androgen antagonists such as abiraterone and enzalutamide [73]. The knockdown of MED12 significantly reduces AR-V7 production and inhibits the expression of the AR target genes, such as prostate-specific antigen (PSA) [74], suggesting that MED12 directly participates in the regulation of expression of the AR target genes. In addition, MED12 knockdown in prostate cancer cells inhibited expression of c-MYC and its downstream proliferation-related genes and reduced the AR-driven tumor growth [74]. Furthermore, MED12 knockdown promotes expression of GLI3 target genes, drives excessive cell growth under castration conditions, and promotes the development of castration resistance through hyperactivation of the GLI3-dependent sonic hedgehog (SHH) pathway [75,76,77].

In uterine leiomyoma cell lines, MED12 knockdown significantly reduces the expression levels of ERα and PR and decreases the proliferation of human leiomyoma cells mediated by the Wnt/β-catenin signaling pathway [78]. Furthermore, MED12 mutants synergize with PR in uterine leiomyoma stem cell proliferation. Interestingly, MED12 is one of the most frequently mutated genes in uterine leiomyomas (approximately 70% of cases). The G44D mutation enhances physical interaction with PR and promotes the binding of MED12 to the receptor activator of NF-κB ligand (RANKL) regulatory region of PR. The RANKL expression level and the stem cell proliferation capacity are significantly higher in the MED12-mutated leiomyoma than in the wild-type MED12 leiomyoma. Hypo-methylated differentially methylated region that is adjacent to the PR binding site (PRBS) of the RANKL gene allows PR/MED12-G44D binding to the PRBS that promotes expression of RANKL and drives the stem cell proliferation and leiomyoma growth through the RANKL–RANK pathway [79,80,81]. In addition, MED12 overexpression interferes with the VDR/BCL6/p53 signaling axis, inhibits the tumor suppressor function of p53, enhances the pro-survival role of BCL6, and drives the malignant progression of glioblastoma [82].

#### 3.4.2. MED13

MED13 has several functional domains, including the N-terminal, PAZ, MID, TRAP240, and PIWI domains [83]. The TRAP240 domain is a critical region for its interaction with transcription factors such as HSI2 [84]. These domains enable MED13 to interact with other proteins and regulate the assembly and activity of the transcription complex. NURR1 is a nuclear receptor that works synergistically with the transcription factor MEF2 to activate expression of the glucose transporter GLUT4 and promote glucose uptake and glycogen storage [85]. In skeletal muscle, MED13 inhibits the activity and expression of NURR1 by either regulating the expression of NURR1 or interacting with the NURR1 protein. Thus, MED13 blocks the synergistic effect of NURR1 with MEF2 and inhibits GLUT4 expression, reduces glucose uptake and glycogen storage, and results in glucose metabolism disorders in skeletal muscle [42,85]. The absence of MED13 in skeletal muscle alleviates the inhibition of the NURR1–MEF2 pathway, reprograms muscle glucose uptake and metabolism, functions as an energy storage ‘buffer’ during caloric excess, and reduces hepatic metabolic burden and lipid deposition [42,85]. In the heart, MED13 downregulates the expression of the target genes of nuclear receptors, such as TR, PPARγ, and RXR [42]. These target genes are involved in diabetes and obesity. The overexpression of MED13 significantly reduced the weight of the high-fat diet mice and increased the energy consumption in the mice [42]. Interestingly, the expression of MED13 is negatively regulated by miR-208a, a heart-specific microRNA, suggesting that MED13 plays a specific role in heart nuclear receptor signaling. The knockout of MED13 in mouse hearts significantly enhanced susceptibility to obesity [42], indicating a crucial role of the heart MED13/nuclear receptor signaling in obesity.

#### 3.4.3. CDK8

CDK8 regulates the nuclear receptor-mediated transcription by facilitating the transcriptional elongation via phosphorylation of the CTD of RNA Pol II [86,87]. In ER-positive breast cancer, CDK8 is recruited to the promoter region of the ERα target gene GREB1 to phosphorylate the S2 site in the RNA Pol II CTD, thereby enhancing transcription elongation efficiency [88]. CDK8 inhibitors, such as Senexin A, significantly suppress ER-induced GREB1 expression, indicating that CDK8 is a modulator of transcriptional activity of ER [88]. It was observed that co-expression of CDK8 and leptin receptor (OBR) exacerbated the malignancy of the ER-negative breast cancer and significantly reduced the overall survival rate of the ER-negative patients who are associated with chemotherapy failure [89], suggesting that CDK8 may play a synergistic role in OBR signaling to promote tumor cell survival and maintain chemoresistance [89].

## 4. Diseases Associated with Interaction of the Mediator Complex with Nuclear Receptors

Based on current research, the Mediator complex is implicated in the pathogenesis of various metabolic diseases, cancers, and developmental abnormalities via specific interactions of its subunits with nuclear receptors (Table 3).

### 4.1. Neoplastic Diseases

#### 4.1.1. Prostate Cancer

MED1 plays a pivotal role in the progression of castration-resistant prostate cancer (CRPC) through its interaction with AR. CDK7-mediated phosphorylation of MED1 facilitates castration resistance by enhancing the AR signaling [92]. Phosphorylation of MED1 by CDK7 increases the binding affinity of the Mediator complex to AR, thereby augmenting transcriptional activity of AR [90,91]. MED1 phosphorylation significantly increases in CRPC and correlates with patient prognosis. Furthermore, the level of MED1 phosphorylation is reversely associated with recurrence-free survival and overall survival [100]. Targeting CDK7 or its substrate MED1 offers a novel therapeutic strategy to overcome the treatment resistance of CRPC. In fact, it has been demonstrated that CDK7 inhibitors, such as THZ1 and CT7001/Samuraciclib, significantly inhibit the AR signaling by blocking MED1 phosphorylation and reducing enzalutamide resistance in preclinical models [101,102].

MED12 was found to be highly expressed in CRPC, and its copy number amplification was associated with enzalutamide resistance. MED12 knockdown induces apoptosis of prostate cancer cells and significantly inhibits the proliferation. CDK8/19 inhibitors (e.g., SEL120-34A) block the kinase activity of the MED12–CDK8 complex, thus reducing the AR target gene expression and enhancing the treatment efficacy of enzalutamide. A combination of CDK8 inhibitors with AR antagonists significantly enhances the inhibition effect of CDK8 inhibitors or AR antagonists alone on the proliferation of CRPC cells [74,78].

#### 4.1.2. Breast Cancer

MED1 is overexpressed in approximately 50% of breast cancers and co-amplified with the HER2 gene [93,94]. Its overexpression promotes tumor growth, metastasis, and cancer stem cell formation by activating the ER and HER2 signaling pathways [95]. Overexpression of MED1 leads to resistance of the ER-positive breast cancer to anti-estrogen drugs such as tamoxifen. Mechanistically, MED1 activates ER transcriptional activity and enhances expression of the pro-proliferative genes, thereby counteracting the drug treatment [96]. Targeting MED1 has been employed in current strategies to overcome breast cancer treatment resistance. These strategies include reducing expression of MED1 by RNA nanotechnology, disrupting interaction with the MED1 functional domains, and combining the MED1 inhibition treatment with conventional therapies. RNA nanotechnology is used to design the RNA nanoparticle that contains both the HER2 RNA aptamer and siMED1 (e.g., pRNA-HER2apt-siMED1) to specifically inhibit both HER2 and MED1. This technology significantly inhibits breast tumor growth, metastasis, and stem cell formation, and enhances the efficacy of tamoxifen [95,96]. Disrupting interaction with the MED1 functional domains mutates the two nuclear receptor-interacting LXXLL motifs of MED1 into LXXAA. When the MED1-LXXAA mutant knockin mice were crossed with MMTV-HER2 transgenic mice, the HER2-driven breast tumor growth and metastasis and cancer stem cell formation were significantly decreased [103]. Furthermore, the hybrid mouse breast tumor cells lost the response to estrogen and decreased expression of the ER/HER2 target genes [103]. These studies indicate that MED1 is an important target for targeted therapy of breast cancer, particularly the HER2-positive breast cancer.

Overexpression of CDK8 is significantly associated with poor prognosis and reduced recurrence-free survival by enhancing estrogen-dependent gene expression in ER-positive breast cancer patients [88]. Furthermore, overexpression of CDK8 drives tumor cell proliferation and promotes breast cancer progression. In HER2-positive breast cancer, high expression of CDK8 is associated with an increased risk of recurrence after treatment, suggesting a role of CDK8 in the development of resistance to targeted therapy [104]. In addition, CDK8 inhibitors can reverse endocrine therapy resistance in ER-positive breast cancer, as demonstrated by the significant inhibition of the Fulvestrant-resistant breast tumor growth with the combination treatment with Senexin B and Fulvestrant [105].

#### 4.1.3. Uterine Fibroids

The MED12 gene is one of the most frequently mutated genes in uterine fibroids, accounting for 70% of all cases [97,98]. The mutations are concentrated at codon 44 of exon 2 (e.g., G44D, G44S), with 42.4% of the G44D mutation [97]. Cross-ethnic studies have shown that MED12 mutations are prevalent among populations in North America, Europe, Asia, and Africa [106]. This provides the patient resources for the development of treatment strategies for uterine leiomyomas. Future research may develop targeted therapies for uterine leiomyomas with the MED12 mutations, such as gene editing-based therapy.

### 4.2. Metabolic Diseases

The Mediator complex, through its subunits such as MED1, MED25, and MED15, interacts with nuclear receptors (e.g., PPARγ, HNF4α, LXR) to participate in the cellular metabolic processes. Abnormal interaction of the Mediator complex with nuclear receptors leads to metabolic diseases. The interaction of the Mediator complex with nuclear receptors not only determines the transcription initiation efficiency but also maintains the cellular metabolic programs through chromatin remodeling and epigenetic modifications. Development of drugs targeting the Mediator complex–nuclear receptor interface (e.g., CDK8 inhibitors, LXXLL motif blocking peptides) offers new precision treatment for metabolic diseases [85].

#### 4.2.1. Diabetes

The interaction of the Mediator complex with nuclear receptors shows significant clinical relevance in diabetes. A mutation in the HNF4α gene is the major monogenic cause of maturity-onset diabetes of the young type 1 (MODY1). Patients exhibit a progressive decline in the insulin secretion capacity of pancreatic β-cells in response to glucose stimulation, along with abnormal birth weight increase and persistent hyperglycemia in adulthood [107]. MED25, as a binding partner of HNF4α, plays an important role in MODY1. Its abnormal function in interaction with HNF4α leads to downregulation of the insulin secretion-related genes (GLUT2, Kir6.2, etc.) and directly causes defects in glucose-stimulated insulin secretion. These data provide a new direction for MODY1 treatment by targeting the LXXLL motif [64].

One of the strategies for insulin sensitization therapy is to target the PPARγ-MED1 pathway. Activation of PPARγ by thiazolidinedione drugs improves insulin resistance through enhancing the expression of adipogenic genes [108]. However, liver-specific knockout of MED1 inhibits PPARγ-mediated adipogenesis, thereby exacerbating the diabetic phenotype [109]. The PPARγ-MED1 pathway is differentially regulated in various tissues, suggesting the PPARγ-MED1 pathway-targeted therapy may be more efficient by targeting the specific organs. These findings have not only defined the central role of the Mediator complex–nuclear receptor interaction in the pathogenesis of diabetes but also revealed the pathogenic mechanism of diabetes, thus establishing a theoretical basis for precise diagnosis and targeted therapy.

#### 4.2.2. Lipid Metabolism Disorders and Obesity

MED1 serves as a key co-activator in PPARγ signaling and shows significant therapeutic potential in high-fat diet-induced fatty liver mouse models [110]. MED1 knockout mice showed a marked alleviation of hepatic lipid accumulation without affecting other metabolic parameters, suggesting that targeting hepatic MED1 may be a strategy for treating non-alcoholic fatty liver disease (NAFLD) [110]. Meanwhile, MED14 functions in regulating adipocyte differentiation and mitigates obesity [110]. Functional loss of MED14 not only inhibits pre-adipocyte differentiation but also improves insulin resistance and dyslipidemia in animal models, providing a new strategy for obesity treatment. It is noteworthy that the tissue-selective intervention strategies for MED1 and MED14 may effectively reduce the risk of systemic side effects and further enhance their clinical value [24].

Overexpression of MED15 has been shown to exacerbate obesity and lipid metabolism disorders induced by high-fat diets through promotion of cholesterol and fatty acid synthesis pathways [52]. Preclinical models have verified MED15 as an obesity drug target. In fact, the small molecule inhibitors targeting MED15-KIX have shown significant improvement in body weight and lipid levels of the diet-induced obese mice. Compound BF175, a boride, inhibits the interaction between MED15-KIX and SREBP1a-TAD, thereby suppressing the expression of genes involved in lipid biosynthesis. In a diet-induced obesity murine model, BF175 reduced hepatic lipid levels and circulating lipid parameters, thus improving lipid homeostasis [99].

## 5. Potential Therapeutic Strategies by Targeting the Interaction Between the Mediator Complex and Nuclear Receptors

The important biological and pathogenic functions of interaction between the Mediator complex and nuclear receptors provide the rationale base for exploring and developing targeted therapeutic strategies for treatment of the Mediator complex/nuclear receptor-associated diseases. Currently, targeting the interaction of the Mediator complex and nuclear receptors for the treatment of diseases is still in an early stage and very few clinical studies have been reported. However, several potential therapeutic strategies have been proposed and explored.

### 5.1. Disrupting the Interaction Interface Between the Mediator Complex and Nuclear Receptors

Strategies disrupting the Mediator complex–nuclear receptor interaction interface primarily include two approaches: competitive inhibitory peptides and small-molecule inhibitors. The design of competitive inhibitory peptides is based on the binding motif LXXLL of the MED1 that interacts with the nuclear receptor AF2 domain (e.g., ERα) [26,34]. Short peptides are designed to mimic this motif peptide sequence, thus competitively disrupting the interaction of MED1 with nuclear receptors. For example, the LXXLL motif peptides targeting ERα significantly suppressed the proliferation of breast cancer cells in vitro by interference with the MED1–ER binding interface [111].

Small-molecule inhibitors targeting the Mediator–nuclear receptor binding interface are identified by high-throughput screening of the chemical compound libraries. For instance, amphiphilic benzene compounds have been identified to mimic the α-helical structure of the LXXLL motif, effectively inhibiting the interaction between ERα and coactivators at low micromolar concentrations and the transcriptional activity [112].

Preclinical studies have demonstrated that small-molecule inhibitors BF175 targeting MED15–KIX interaction significantly improved body weight and lipid profiles in murine obesity models [99]. However, the effect of BF175 on obesity remains in the preclinical phase, and its pharmacological properties, including selectivity and off-target effects, as well as long-term safety, have not been thoroughly evaluated. The current findings are exclusively derived from murine models.

### 5.2. Targeting the Cofactors That Mediate Interaction Between the Mediator Complex and Nuclear Receptors

Nuclear receptors indirectly recruit the Mediator complex via cofactors such as SRC, CoCoA, and CCAR1. These adaptor proteins facilitate transcriptional activation by binding to the Mediator complex [113,114,115]. Consequently, disrupting the interaction between adaptor proteins and Mediator can interfere with transcriptional activation. For example, specifically targeting the LXXLL motif of SRC can diminish the Mediator recruitment and subsequent transcriptional activity. This approach has been validated in breast cancer cells and shown that targeting the SRC family proteins effectively inhibits estrogen receptor-mediated gene transcription [116,117].

### 5.3. Targeting the CDK8 Kinase Module

The CDK8 kinase module represents a promising therapeutic target. This module dynamically interacts with the Mediator complex core and modulates the function of the Mediator complex-regulated nuclear receptor signaling. Dysregulation of this module promotes tumorigenesis [5,118]. Therefore, disrupting the CDK8–Mediator complex interface or developing kinase inhibitors could selectively abrogate the nuclear receptor-associated oncogenic pathways [88]. Several CDK8/CDK19 inhibitors, including Cortistatin A, Senexin, and CCT251545, have been identified [119].

Although the aforementioned strategies exhibit therapeutic potential, the application of these strategies for clinical treatment has not been established. Future clinical research may follow these strategies and develop the drugs that specifically target the interaction between the Mediator complex and nuclear receptors.

## 6. Conclusions and Perspectives

The Mediator complex serves as a central hub for transcriptional regulation in eukaryotic organisms and integrates transcriptional signals through its modular structure, thereby precisely coordinating gene expression. The nuclear receptors, functioning as ligand-dependent transcription factors, play important roles in every aspect of cellular activities. The interaction of the Mediator complex with nuclear receptors translates the nuclear receptor signals into gene expression output and biological function. Dysregulation of the interaction of the Mediator complex with nuclear receptors causes multiple diseases, such as cancer, diabetes and obesity. Thus, further revealing the mechanism underlying the regulation of transcriptional activity of nuclear receptors by the Mediator complex is crucial for us to understand the pathological causes of both the Mediator complex- and nuclear receptor-associated diseases and develop therapeutic strategies.

Future research may need to address the specificity of the interaction between the Mediator complex and nuclear receptors and understand the precise regulatory mechanism by which the Mediator complex regulates the nuclear receptor transcriptional output, particularly regarding the functional specificity of the nuclear receptors across various tissues and cell types. In translational medicine, dual-track therapeutic strategies may be developed, such as those targeting both the Mediator complex and nuclear receptors. Furthermore, integrating AI-predicted protein interaction networks with the CRISPR-Cas high-throughput screening system may systematically identify key nodes in the Mediator complex–nuclear receptor interaction-associated cellular signaling network. These AI tools may also be utilized to mine clinical data for optimizing personalized treatment regimens regarding the Mediator complex and nuclear receptor-associated diseases. Through investigation of the interaction of the Mediator complex with nuclear receptors and discovery of the mechanism by which the Mediator complex controls the nuclear receptor transcriptional activity, we may develop novel targeted therapeutic strategies for precision treatment of cancer, diabetes, obesity and other diseases.

## Figures and Tables

**Table 1:** Composition of the human Mediator complex.

Module	Subunit
Head	MED6
	MED8
	MED11
	MED14
	MED17
	MED18
	MED20
	MED22
	MED27
	MED28
	MED29
	MED30
Middle	MED1
	MED4
	MED7
	MED9
	MED10
	MED19
	MED21
	MED26
	MED31
Tail	MED15
	MED16
	MED23
	MED24
	MED25
CDK8-Kinase module	MED12/12L
	MED13/13L
	CDK8/CDK19
	CycC

**Table 2:** NRs and their directly interacting Mediator subunits.

Subunit	NR	Mediator Subunit Domains Involved	NRs Domains Involved	Reference
MED14	GR		AF1	[29]
	PPARγ			[30]
	HNF4			[31]
	ERα			[32]
MED1	AR	LXXLL	AF1/AF2	[33]
	ER			[26,34]
	PPARγ			[35]
	GR			[36]
	TR			[34]
	VDR			[37]
	RAR			[38]
	RXR			[38]
	HNF4			[31]
MED15	NHR-49	KIX	LBD	[39]
MED25	RAR	LXXLL	AF2	[40]
	HNF4α			[41]
MED13	NURR1			[42]

**Table 3:** Mediator subunit involvement in NR-associated diseases.

Disease	Subunit	Molecular Mechanism	Preclinical Features	Reference
Prostate cancer	MED1	Phosphorylation modification	Cancer progression propensity	[90,91,92]
	MED12	Overexpression	Drug resistance	[74,78]
Breast cancer	MED1	Overexpression	Promotes cancer progression	[93,94]
			Drug resistance	[95,96]
	CDK8		Promotes cancer progression	[88]
Uterine Fibroids	MED12	Gene mutations	Promotes tumor growth	[97,98]
Diabetes	MED25	Gene mutations	Insulin secretion defect	[64]
Lipid Metabolism disorders and Obesity	MED15	Overexpression	Lipid metabolism disorders	[99]

## Data Availability

Not applicable.
